# Dataset on child vaccination in Brazil from 1996 to 2021

**DOI:** 10.1038/s41597-023-01939-0

**Published:** 2023-01-11

**Authors:** Patricia de Moraes Mello Boccolini, Cristiano Siqueira Boccolini, Laís de Almeida Relvas-Brandt, Ronaldo Fernandes Santos Alves

**Affiliations:** 1 Institute of Scientific and Technological Communication and Information in Health, Oswaldo Cruz Foundation, Rio de Janeiro, RJ Brazil; 2 Center for Information, Public Policies and Social Inclusion, NIPPIS, Petropolis Medical School, UNIFASE, Petrópolis, RJ Brazil

**Keywords:** Epidemiology, Health care

## Abstract

We present a machine-readable and open-access dataset on vaccination results among children under five years old in Brazil from 1996 to 2021. This dataset is interoperable with epidemiological data from the VAX*SIM project and reusable by the research community worldwide for other purposes, such as monitoring vaccination coverage and studying its determinants and impacts on child morbidity and mortality. The dataset gathers official and public information from the Brazilian National Immunisation Program, the Institute of Geography and Statistics, the Institute for Applied Economic Research, and the Ministry of Health. It includes 2,442,863 observations and 35 attributes aggregated by years, policy-relevant geographic units (country, macroregions, states, municipalities, and capitals), and age groups on 1,344,480,329 doses of 28 vaccines aimed to prevent 15 diseases, estimates of their target-population coverage, indicators of the vaccination coverage’s homogeneity, dropout rates, and spatial, demographic, and socioeconomic data. We automated all data processing and curation in the free and open software R. The codes can be audited, replicated, and reused to produce alternative analyses.

## Background & Summary

Vaccination is a cost-effective intervention for child health, reducing illness and death from vaccine-preventable diseases. Immunisation Information Systems (IIS) are confidential computerised, population-based databases that record all vaccination doses administered by health care providers to people residing in a given location^1^.

The Brazilian National Immunisation Program (PNI) was created in 1973 by the Ministry of Health. Due to the expansion of vaccination coverage and the number of immunizers offered by PNI in the 1980s^2^, the data processing and calculation of vaccination indicators were saturated in Brazil since they were recorded in spreadsheets and manual forms^1,2^. In 1994, the Ministry of Health started the development of the National Immunisation Program Information System (SIPNI), aggregating the existing national vaccination subsystems^3^. The SIPNI went through several updates, was consolidated in 2008, and has vaccine records for the entire Brazilian population^3^.

The complete “raw” SIPNI databases are not publicly available. For this study, we requested the aggregated dataset from the Ministry of Health (MoH) through the National Access to Information Law 12527/2011^4^, but we were denied. The MoH suggested we do tabular queries on the governmental website (TABNET) managed by the Department of Informatics of Brazil’s Unified Health System (DATASUS)^5^ to access the aggregated data. However, building a machine-readable dataset for the entire 25 years of data available would require thousands of manual tabular queries on the TABNET, followed by the organisation and junction of the obtained spreadsheets. This manual process would increase the occurrence of errors and has a very high operational cost, making it virtually infeasible to get a complete historical vaccination series in Brazil.

Therefore, this paper innovates by offering technological solutions for data extraction, transformation, and loading (ETL) and integration, harmonisation, and enrichment (IHE). We created an automated routine to extract, process, and curate the SIPNI data from the TABNET tabulator, allowing the construction of a single database on vaccination aggregated by age groups under five years old, calendar years, and Brazilian policy-relevant geographic units.

Vaccination data spans over 25 years. The data extraction, curation, and enrichment codes will be automatically executed yearly in March of the subsequent year, updating the dataset in the same format. Eventually, the extraction code can be reprogrammed to adapt to possible TABNET tabulation format changes providing continuous and sustainable data on childhood vaccination from SIPNI.

The dataset has information on doses of 28 vaccines designed to protect children under five years old against 15 diseases. The dataset also includes estimates of the size of the target population, indicators of vaccination coverage, homogeneity, and dropout, as well as spatial, demographic, and socioeconomic data.

This dataset is interoperable with epidemiological data from two major studies^6,7^, including avoidable child mortality, hospitalisation, primary healthcare, and others. Most notably, it was developed in the framework of the study “The role of social media, Bolsa Familia Program (BFP), and Primary Health Care (PHC) in vaccination coverage for children under five in Brazil” (VAX*SIM), which aims to understand and analyse the determinants of the evolution of vaccination coverage in Brazil. The study uses a theoretical and conceptual matrix to assess the association between vaccination coverage in Brazilian municipalities with socioeconomic factors, the PHC and BFP coverage, and vaccine-preventable child morbidity and mortality.

### Data description

**Fig. 1:**
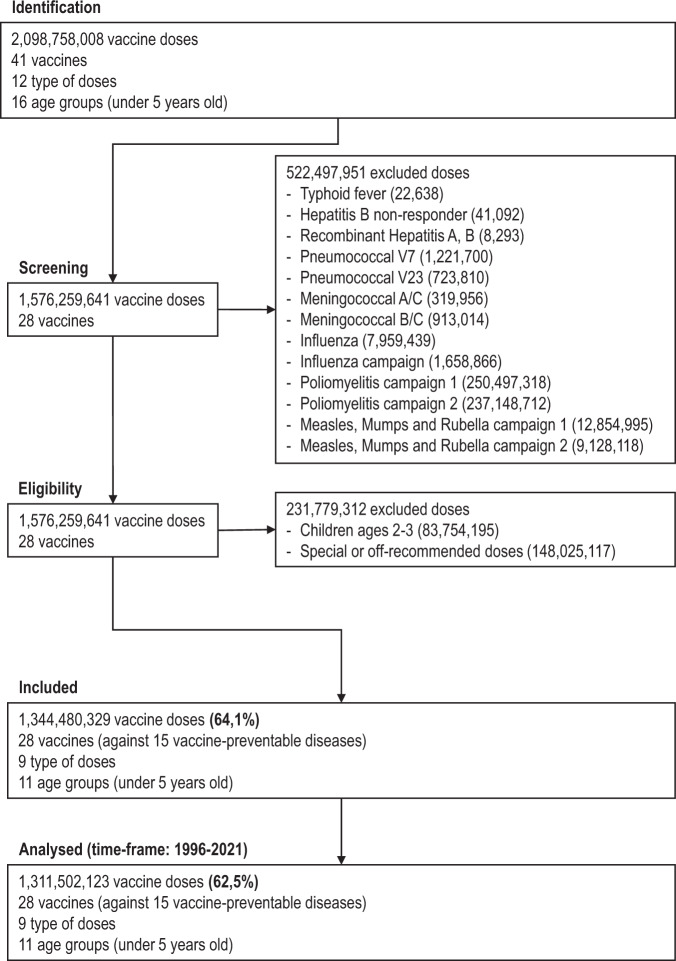
Flow diagram for the dataset on child vaccination in Brazil from 1996 to 2021.

The dataset covered 1,344,480,329 doses of 28 vaccines to protect against 15 diseases. It also includes counts of target populations’ estimates and indicators of vaccination coverage, homogeneity, and dropout rate. We add previously consolidated spatial, demographic, and socioeconomic data from the Brazilian Institute of Geography and Statistics (IBGE), Institute for Applied Economic Research (IPEA), and Ministry of Health (MoH), which are described and available elsewhere^8^. Figure 1 presents a flow diagram for the dataset on child vaccination in Brazil from 1996 to 2021. Regarding the exclusion criteria, we did not consider the vaccines that do not compose the childhood’s routine immunisation schedule. We also excluded the Polio, Measles, Mumps and Rubella doses during the campaigns because they are counted in the total doses applied during the year, composing the Polio vaccination coverage. Any applied doses in children between 2 and 3 years old are considered late in the Brazilian official vaccination scheme. Therefore, we have excluded this age group from our dataset.

## Methods

### Data construction: Workflow

**Fig. 2:**
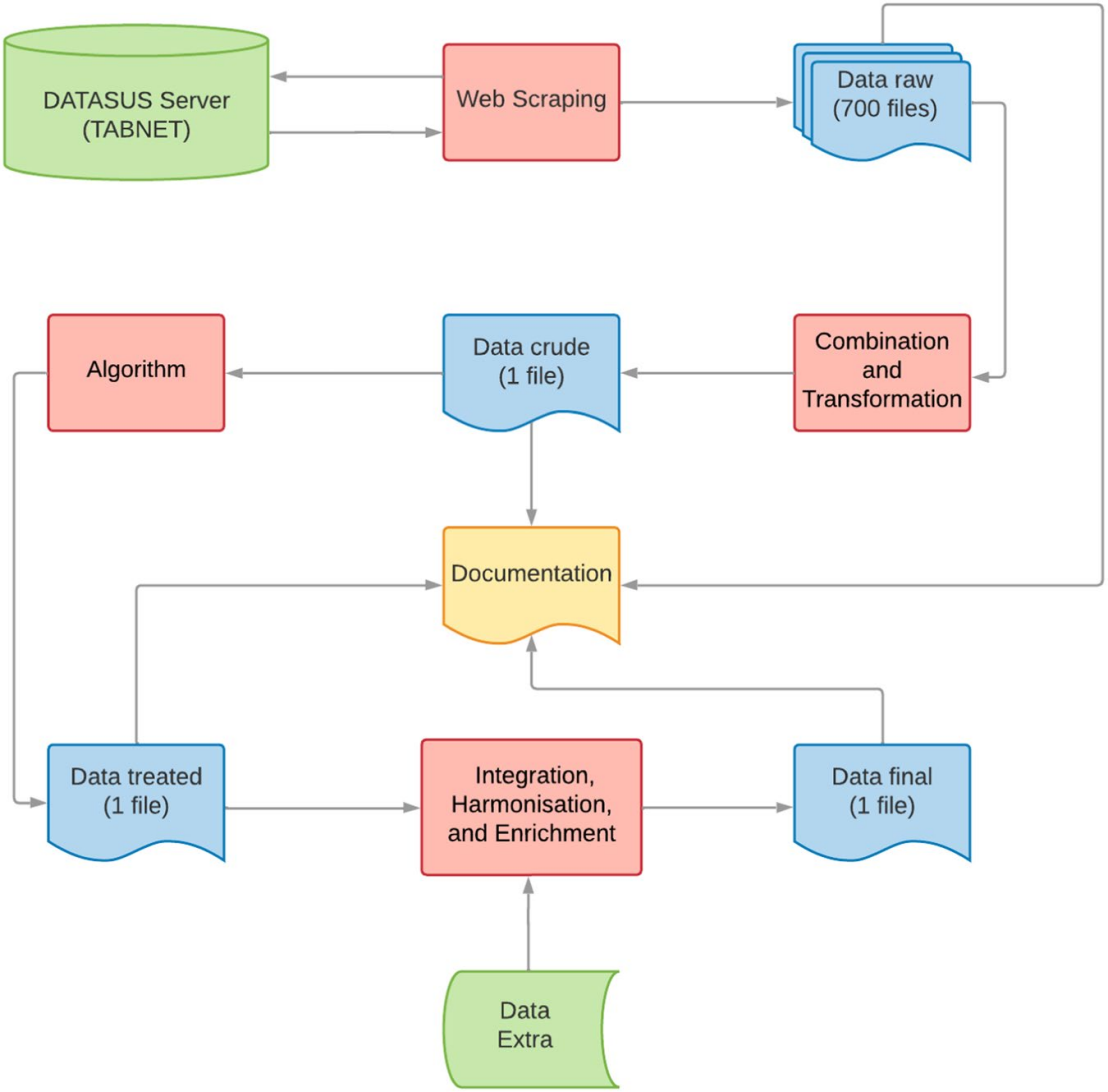
Workflow for vaccination database, Brazil, 1996–2021.

The data workflow comprises five main steps (Fig. 2). The first step covered mapping the availability of data on administered doses of vaccines from the Brazilian childhood’s routine immunisation schedule in the TABNET application^5^. TABNET is a generic public domain tabulator that allows you to organise information from Brazil’s national health system (SUS) databases, including SIPNI. We checked data availability on 41 vaccines, 12 types of doses, and 16 age groups of children under five years for the 5,570 municipalities in the country from 1996 to 2021. Such resulted in 10,530 queries to the TABNET.

The second step concerned web scraping data from the requests identified as valid in the previous step, which resulted in 700 separate raw files. We programmatically extracted the vaccination data on March 23, 2022. The third stage covered the data combination and transformation processes, whose main characteristics were:

1. Variables renaming and observations filtering.
2. Correction of codes and names identifying geographic units.
3. Age groups’ recategorization.
4. Cleansing numeric values, e.g., excluding special characters.
5. Excluding vaccine doses not included in the Brazilian childhood’s routine immunisation schedule and vaccine doses included in the regular vaccination schedule administered in a particular situation or off-recommended age groups.
6. Enrichment of the municipal datasets with data aggregated by states, macroregions, and national levels.

The fourth step involved the application of business rules for the construction of vaccination indicators, which combine groups of vaccines with different formulations for the same disease (Tables 1, 2).

**Table 1:** Rules for measuring the vaccination coverage indicators of routine immunisation schedule for children under one-year-old. Brazil, 1996–2021.

Indicators*	Vaccines	Immunisation	Time Frame
**Under 30 days old**			
Hepatitis B	HepB	1st dose	1996
**Under 1 year old**			
Measles	Measles, monovalent	Single dose	1994–2003
Yellow fever	Yellow fever	Single dose	1994
BCG	BCG	Single dose	1994
	BCG-Hansen	Single dose	1994
Diphtheria and Tetanus**	DT	3rd dose	1994
Diphtheria, Tetanus and Pertussis	DTP	3rd dose	1994
	DTaP	3rd dose	2000
	DTP/Hib	3rd dose	2002
	DTP/Hib/HepB	3rd dose	2003
	DTaP/Hib/HepB/IPV	3rd dose	2006
	DTaP/Hib/IPV	3rd dose	2013
Poliomyelitis	OPV	3rd dose	1994
	IPV	3rd dose	2000
	DTaP/Hib/HepB/IPV	3rd dose	2006
	IPV/OPV	3rd dose	2012
	DTaP/Hib/IPV	3rd dose	2013
Hepatitis B	HepB	3rd dose	1996
	DTP/Hib/HepB	3rd dose	2003
	DTaP/Hib/HepB/IPV	3rd dose	2006
*Haemophilus influenzae* type B	Hib	3rd dose	2000
	DTP/Hib	3rd dose	2002
	DTP/Hib/HepB	3rd dose	2003
	DTaP/Hib/HepB/IPV	3rd dose	2006
	DTaP/Hib/IPV	3rd dose	2013
Rotavirus	RV1	2nd dose	2006
	RV5	3rd dose	2013
Pneumococcal	PCV10	2nd dose	2010
	PCV13	2nd dose	2010
Meningococcal C	MenC	2nd dose	2010
	MenACWY	2nd dose	2017

HepB: Hepatitis B. BCG: Bacille Calmette-Guerin. BCG-Hansen: Bacille Calmette-Guerin for leprosy contacts. DT: Diphtheria and Tetanus. DTP: Diphtheria, Tetanus and Pertussis. DTaP: Diphtheria, Tetanus, and acellular Pertussis. Hib: Haemophilus influenzae type B. IPV: Inactivated poliovirus vaccine. OPV: Oral poliovirus vaccine. IPV/OPV: Sequential inactivated and oral poliovirus vaccines. RV1: Rotavirus vaccine monovalent. RV5: Rotavirus vaccine pentavalent. PCV10: Pneumococcal vaccine 10-valent. PCV13: Pneumococcal vaccine 13-valent. MenC: Meningococcal C. MenACWY: Meningococcal ACWY. MR: Measles and Rubella. MMR: Measles, Mumps, and Rubella. MMRV: Measles, Mumps, Rubella, and Varicella. HepA: Hepatitis A. VAR: Varicella. *Immunisation coverage goal: 100% for yellow fever. 90% for BCG and rotavirus. 95% for other diseases. **The measurement of vaccination coverage indicators for these diseases should include all antigen-specific containing vaccines for these diseases.

**Table 2:** Rules for measuring the vaccination coverage indicators of routine immunisation schedule for children at one and four years old. Brazil, 1996–2021.

**1 year old**			
Measles and Rubella**	MR	Single dose	2001–2004
Measles, Mumps and Rubella (1)	MMR	1st dose	2000
	MMRV (quadruple)	1st dose	2016
Measles, Mumps and Rubella (2)	MMR	2nd dose	2000
	MMRV (quadruple)	2nd dose	2016
	MMRV (tetra)	1st dose	2013
Varicella	VAR	1st dose	2013
	MMRV (quadruple)	1st dose	2016
	MMRV (tetra)	1st dose	2013
Hepatitis A	HepA	Single dose	2014
Diphtheria, Tetanus and Pertussis	DTP	1st booster dose	1994
	DTaP	1st booster dose	2000
Poliomyelitis	OPV	1st booster dose	1994
	IPV	1st booster dose	2000
	IPV/OPV	1st booster dose	2012
Pneumococcal	PCV10	Booster dose	2010
	PCV13	Booster dose	2010
Meningococcal C	MenC	Booster dose	2010
	MenACWY	Booster dose	2017
**4 years old**			
Diphtheria, Tetanus and Pertussis	DTP	2nd booster dose	1994
	DTaP	2nd booster dose	2000
Poliomyelitis	OPV	2nd booster dose	1994
	IPV	2nd booster dose	2000
	IPV/OPV	2nd booster dose	2012
Varicella	VAR	2nd dose	2013
Yellow fever	Yellow fever	Booster dose	1994

HepB: Hepatitis B. BCG: Bacille Calmette-Guerin. BCG-Hansen: Bacille Calmette-Guerin for leprosy contacts. DT: Diphtheria and Tetanus. DTP: Diphtheria, Tetanus and Pertussis. DTaP: Diphtheria, Tetanus, and acellular Pertussis. Hib: Haemophilus influenzae type B. IPV: Inactivated poliovirus vaccine. OPV: Oral poliovirus vaccine. IPV/OPV: Sequential inactivated and oral poliovirus vaccines. RV1: Rotavirus vaccine monovalent. RV5: Rotavirus vaccine pentavalent. PCV10: Pneumococcal vaccine 10-valent. PCV13: Pneumococcal vaccine 13-valent. MenC: Meningococcal C. MenACWY: Meningococcal ACWY. MR: Measles and Rubella. MMR: Measles, Mumps, and Rubella. MMRV: Measles, Mumps, Rubella, and Varicella. HepA: Hepatitis A. VAR: Varicella. *Immunisation coverage goal: 100% for yellow fever. 90% for BCG and rotavirus. 95% for other diseases. **The measurement of vaccination coverage indicators for these diseases should include all antigen-specific containing vaccines for these diseases.

The fifth step in the workflow involved data integration, harmonisation, and enrichment. We linked the dataset produced in the previous step with a spatial, demographic, and socioeconomic dataset^8^ according to the years and codes of the geographic units. It includes geocoding of municipality centroids, total population size, child population by age group, birth and mortality measures, Brazilian Municipal Human Development Index (MHDI), Gini coefficient, Gross Domestic Product (GDP), and sanitation. Furthermore, we created the following variables: (i) vaccination dropout number (number of children unvaccinated with final dose in multi-dose schedules; i.e., the difference between the number of last doses and the number of initial doses); (ii) vaccination dropout rate (vaccination dropout number per number of initial doses); (iii) vaccination coverage (number of final vaccine doses in single and multi-dose schedules per the estimated target population); and (iv) vaccination coverage homogeneity (proportion of municipalities that met the vaccination coverage goal in Brazil, macroregions, and states). R codes and data processing/curation were peer-reviewed, and their results were compared to the information on official sites.

## Data Records

We provide a machine-readable and open-access dataset on Brazilian National Immunisation Program (PNI)^5^ vaccination results in children under five from 1996 to 2021. The dataset has 2,442,863 observations and 35 attributes aggregated by years (1996–2021), policy-relevant geographic units (country, macroregions, states, municipalities, and capitals), and age groups (children under one-year-old, and four years old). Table 3 describes the key details of this dataset.

**Table 3:** Key details about the dataset.

Domains	Sources	Time frames	Number of variables	Measurements
**Basic information**				
Geographical	IBGE	1996–2021	5	Scope, names, and codes of geographical units; latitude/longitude of municipalities centroids
Temporal	—	1996–2021	1	Years
**Additional information**				
Demographic	IBGE	1996–2021	1	Total population size
	IBGE, MoH (Sinasc)	1996–2021	1	Birth rate
Vital statistics	MoH (Sinasc)	1996–2021	1	Number of live births
	MoH (SIM)	1996–2021	2	Number of all deaths and deaths in children under 1-year-old
	MoH (Sinasc, SIM)	1996–2021	2	Crude, and infant mortality rate
Socioeconomic	IBGE	1999–2019	3	Gini coefficient; GDP total and per capita (current R$)
	IPEA	1991, 2000, 2010	5	Brazilian MHDI global, education, longevity, and income; inadequate water supply and sanitation
**Principal information**				
Demographic	MoH (Estimates, Sinasc, SIM)	1996–2021	2	Age groups and child population estimates by age-groups
Immunisation	SIPNI	1996–2021	12	Vaccination indicators, doses, coverage, dropout, and homogeneity

IBGE: Institute of Geography and Statistics. MoH: Ministry of Health. Sinasc: Live Birth Information System. SIM: Mortality Information System. IPEA: Institute for Applied Economic Research. SIPNI: National Immunisation Program Information System. Notes: We collected the spatial data from IBGE Brazilian Territorial Division (2020) and the total population data from IBGE demographic censuses (2000, 2010), inter-census counts (1996, 2007), and population estimates (other years).

The data resources described in this paper are freely and openly available on the Synapse repository at 10.7303/syn26453964^9^. The Synapse is a collaborative workspace for reproducible data-intensive research projects, which supports the integrated presentation of datasets, codes, and documentation, fine-grained access control, and provenance tracking. Anyone can browse the content on the Synapse website, but you must register for an account using your email address to download the files and datasets. The download is possible using web or programmatic clients, such as R and Python^10^.

Table 4 provides an overview of the files and datasets stored in Synapse. We automated all data processing and curation in the free and open software R. Data files 1–3 hold the codes for extraction, transformation, and loading routines. We extracted the data in its original format (dataset 1) and separately saved each workflow endpoint’s processed data (datasets 2–4). Data file 4 builds the final dataset (datasets 5 and 6), which was integrated, harmonised, and enriched with spatial, demographic, and socioeconomic data^8^. All R codes can be audited, replicated, and reused to produce alternative analyses.

**Table 4:** Overview of data files and datasets.

Label	Name of datafile/datasets	File type (file extension)	Data repository and identifier
Data file 1	script_sipni_webscraping	R code (.r)	Synapse: 10.7303/syn26453964^9^
Data file 2	script_sipni_ingestion	R code (.r)	Synapse: 10.7303/syn26453964^9^
Data file 3	script_sipni_algorithm	R code (.r)	Synapse: 10.7303/syn26453964^9^
Data file 4	script_master_immunisation	R code (.r)	Synapse: 10.7303/syn26453964^9^
Data file 5	sipni_sprint_dataselfie	HTML (.html)	Synapse: 10.7303/syn26453964^9^
Data file 6	sipni_algorithm_dataselfie	HTML (.html)	Synapse: 10.7303/syn26453964^9^
Data file 7	immunisation_master_dataselfie	HTML (.html)	Synapse: 10.7303/syn26453964^9^
Data file 8	sipni_ws_control	excel (.xlsx)	Synapse: 10.7303/syn26453964^9^
Data file 9	sipni_info_data_raw	excel (.xlsx)	Synapse: 10.7303/syn26453964^9^
Data file 10	immunisation_master_overview	excel (.xlsx)	Synapse: 10.7303/syn26453964^9^
Data file 11	R Shiny App	Webpage (https)	Synapse: 10.7303/syn26453964^9^
Dataset 1	sipni_data_raw	zipped (.zip)	Synapse: 10.7303/syn26453964^9^
Dataset 2	sipni_crude_data	R data (.rdata)	Synapse: 10.7303/syn26453964^9^
Dataset 3	sipni_clean_data	R data (.rdata)	Synapse: 10.7303/syn26453964^9^
Dataset 4	sipni_final_data	R data (.rdata)	Synapse: 10.7303/syn26453964^9^
Dataset 5	immunisation_master_data.RData	R data (.rdata)	Synapse: 10.7303/syn26453964^9^
Dataset 6	immunisation_master_data.csv	delimited text (.csv)	Synapse: 10.7303/syn26453964^9^

Notes: Anyone can browse the content on the Synapse website, but you must register for an account using your email address to download the files and datasets.

The HTML files show type-specific information for all intermediate and final datasets attributes, including statistical summaries and missing frequencies (data files 5–7). Data file 8 includes information about requests made to the TABNET website, and data file 9 contains the responses to the web scraping. Data file 10 documents the metadata and attribute descriptions of the final dataset (datasets 5 and 6). Data file 11 presents mapping and time trends of vaccination results.

Remarkably, the final dataset (datasets 5 and 6) gathered 35 attributes described in the dictionary of terms (data file 10), including spatial, demographic, socioeconomic data, birth and mortality measures, as well as administered dose counts, measurements of coverage, dropout, and homogeneity, and target population sizes according to age groups for 24 vaccination indicators – defined as vaccine groups with different formulations against the same diseases.

## Technical Validation

Initially, we mapped the availability of vaccination data in children under five years on the TABNET website, considering all possible combinations between the variables of interest (municipalities, years, vaccines related to the Brazilian routine vaccination schedule, types of administered doses, and age groups). Subsequently, we checked the requests which do not return valid results through new programmatic queries to the TABNET. Besides, we compared the results obtained from successful requests with those presented in TABNET, considering the absolute numbers of administered doses and the relative estimates of vaccination coverage. All R codes for data extraction, processing, and curation were peer-reviewed. We also performed a time-trend analysis of vaccination indicators to detect abnormalities and inconsistencies.

## Usage Notes

We should note the usages and warnings of the final dataset. First, it has a mixed ecological structure in which observation units are geographically defined populations at different time points. This structure allows interoperability with epidemiological data from the VAX*SIM project – whose main objectives are monitoring vaccination coverage among children under five years in Brazil and studying its determinants and impacts on child morbidity and mortality. The research community worldwide can use the dataset for other purposes, such as health inequality studies, multilevel analysis, and cross-country comparisons of vaccination results.

Additionally, it can be a valuable dataset for Brazilian health managers and professionals to evaluate compliance with vaccination goals, build data visualisation dashboards, and formulate programs or policies aimed at regions with lower vaccination coverage rates. Second, our vaccination indicators combined the administered doses of all antigen-specific vaccines for the same diseases. For example, we calculated poliomyelitis vaccination coverage among children under one year by the ratio between the sum of 3rd doses of five vaccines (OPV, IPV, DTaP/Hib/HepB/IPV, IPV/OPV, and DTaP/Hib/IPV) and the size of the target population.

It is a usual strategy to monitor vaccination indicators by immunising type (e.g. Polio, BCG, and MR) to avoid possible variations in the routine vaccination that group or replace vaccines over time. Regarding vaccination coverage, the indicators are based on the age at which children should receive the vaccine. Thus, we choose not to account for vaccinated children outside the usual schedule, excluding, in this dataset, the vaccine applied outside the recommended age group. Besides, the vaccination data refers to the application sites, not necessarily the children’s place of residence.

**Fig. 3:**
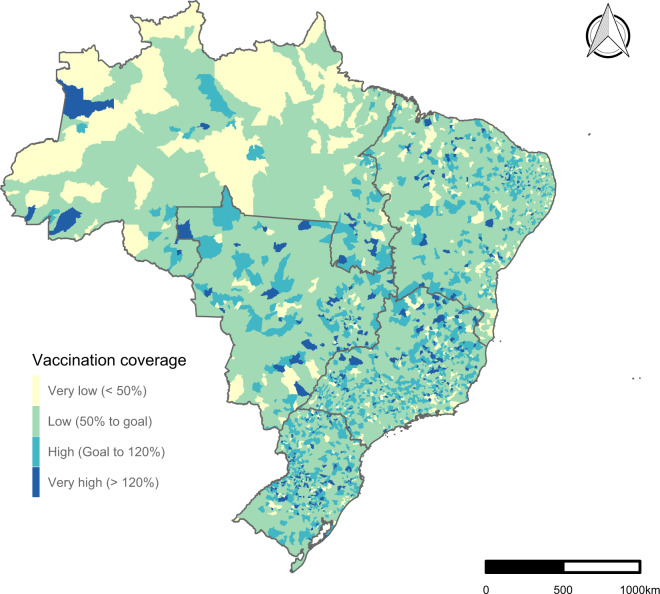
Polio vaccination coverage for children under one year old among Brazilian municipalities in 2021.

**Fig. 4:**
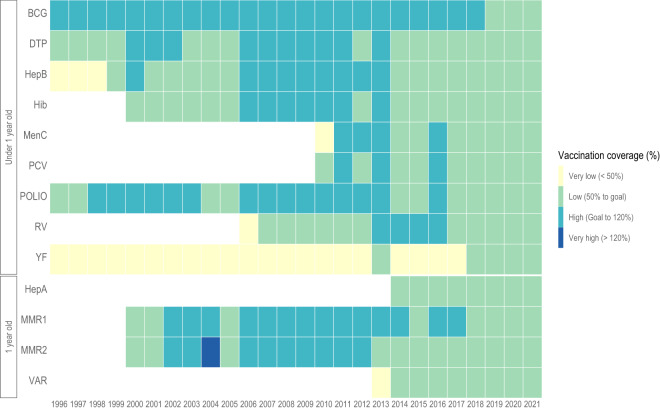
Time-trend of vaccination coverage in Brazil for children at one year old and under one year.

Third, while immunising 100% of the target population is theoretically possible, especially in small cities, true immunisation levels of 100% are unlikely. We observed coverage levels exceeding 100% in the dataset, likely due to systematic errors in the ascertainment of the numerator or denominator, mid-year changes in target age groups, or the inclusion of children from other cities in the numerator^11^. Therefore, to analyse the data, we suggest categorising the vaccination coverage in Very Low (<50%), Low (50 to Goal%), High (Goal to 120%) and Very High (>120%), according to Braz *et al*. (2016) classification^12^ (Figs. 3, 4). We also fixed the maximum values at 150% to avoid implausible outliers.

Fourth, our vaccination relative coverage among children of one year (2006-onwards) considered the total live births minus the infant deaths in the previous year and may diverge somewhat from MoH estimates, which consider only the number of live births in the last year. Finally, we could only build the dataset at an ecological level, including the administered doses in public and private health services, but it was impossible to separate the dose counts of these two sectors. Our data extraction and processing routines are sustainable and automatic, and we intend to update this dataset annually. Figure 3 is an example of how to apply the data to describe the polio vaccination coverage among Brazilian municipalities in 2021, and Fig. 4 is another example of how to apply the data to describe the time-trend of vaccination coverage in Brazil for children at one year old and under one year.

## Data Availability

We automated all data processing and curation in the free and open software R (4.2.1, current version for windows). The data resources described in this paper, including R codes, can be accessed with no restrictions on the Synapse repository at 10.7303/syn26453964. Anyone can browse the content on the Synapse website, but you must register for an account using your email address to download the files and datasets. Please see Table 3 and references^6,7,9^ for details and links to the data resources.
